# FTO influences adipogenesis by regulating mitotic clonal expansion

**DOI:** 10.1038/ncomms7792

**Published:** 2015-04-17

**Authors:** Myrte Merkestein, Samantha Laber, Fiona McMurray, Daniel Andrew, Gregor Sachse, Jeremy Sanderson, Mengdi Li, Samuel Usher, Dyan Sellayah, Frances M. Ashcroft, Roger D. Cox

**Affiliations:** 1 MRC Harwell, Mammalian Genetics Unit, Harwell Oxford OX11 0RD, UK; 2 Department of Physiology, Anatomy and Genetics, University of Oxford, Oxford OX1 3PT, UK

## Abstract

The fat mass and obesity-associated (*FTO*) gene plays a pivotal role in regulating body weight and fat mass; however, the underlying mechanisms are poorly understood. Here we show that primary adipocytes and mouse embryonic fibroblasts (MEFs) derived from *FTO* overexpression (*FTO-4*) mice exhibit increased potential for adipogenic differentiation, while MEFs derived from *FTO* knockout (*FTO-KO*) mice show reduced adipogenesis. As predicted from these findings, fat pads from *FTO-4* mice fed a high-fat diet show more numerous adipocytes. *FTO* influences adipogenesis by regulating events early in adipogenesis, during the process of mitotic clonal expansion. The effect of *FTO* on adipogenesis appears to be mediated via enhanced expression of the pro-adipogenic short isoform of RUNX1T1, which enhanced adipocyte proliferation, and is increased in *FTO-4* MEFs and reduced in *FTO-KO* MEFs. Our findings provide novel mechanistic insight into how upregulation of *FTO* leads to obesity.

## Abstract (web summary)

Mutations in the *FTO* gene have been linked to obesity. Here, Merkestein *et al.* provide *in vitro* and *in vivo* evidence that *FTO* directly regulates adipogenesis in mice at the stage of mitotic clonal expansion, likely by modulating the expression of the transcription factor RUNX1T1.

## Graphical abstract

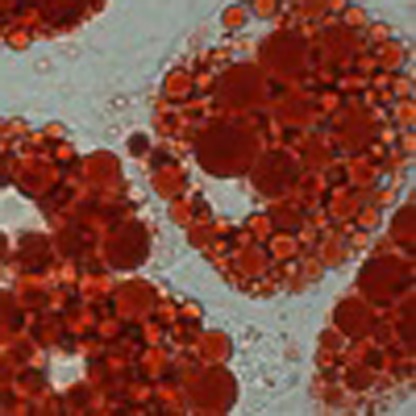

The fat mass and obesity-related gene (*FTO*) was the first gene shown to play a role in common obesity. Genome-wide association studies identified a single-nucleotide polymorphism (SNP) in the first intron of the *FTO* gene that is associated with body mass index in Caucasians^1^, and subsequent studies confirmed this finding in multiple human populations and ethnic groups^2^. The increase in body mass index results almost entirely from an increase in fat mass^3^,^4^,^5^,^6^. *In vivo* studies using mouse models have also demonstrated that knockout of *FTO*^7^,^8^,^9^, or a mutation in *FTO* that impairs its function^10^, leads to reduced body weight and fat mass. Conversely, overexpression of *FTO* results in increased body weight and fat mass^11^. Recently, it has been proposed that the obesity-related SNPs in *FTO* influence obesity susceptibility not by affecting *FTO* gene expression, but by altering the expression of the adjacent genes *IRX3* and *RPGRIP1L*^12^,^13^. Other studies, however, have linked the SNP risk genotype to increased *FTO* expression in human fibroblasts and blood cells^14^,^15^. Furthermore, the mouse data strongly support a role for *FTO* in regulating body weight and fat mass, and a mutation in the catalytic domain of *FTO* (R316Q) in humans results in a severe phenotype accompanied by growth retardation^16^. Thus, although the intronic SNPs may operate via different mechanisms, *FTO* clearly plays a role in the regulation of fat mass.

The mechanism by which *FTO* affects fat mass has been elusive. *FTO* is a nucleic acid demethylase that removes methyl groups from both DNA and RNA^17^,^18^,^19^. It is commonly thought that its most important functional role is demethylating N^6^methyladenosine (m^6^A)^19^, which thereby could regulate processing, stability and alternative splicing of mRNAs^20^,^21^,^22^. A recent study of 3T3-L1 cells supports this idea, showing that *FTO* controls mRNA splicing by regulating the ability of the splicing factor SRSF2 to bind mRNA in an m^6^A-dependent way^22^. One of the targets of SRSF2 is Runt-related transcription factor 1 (RUNX1T1), an adipogenesis-related transcription factor that exists in two splice variants, a long (L) and a short (S) isoform. Overexpression of the S isoform of RUNX1T1 in 3T3-L1 cells stimulates adipogenesis, suggesting that *FTO* might act via RUNX1T1 to enhance adipocyte formation^22^. We therefore explored whether *FTO* modulates adipogenesis in native cells derived from mice overexpressing *FTO*^11^ or in which *FTO* had been deleted^8^. Our data provide firm evidence that *FTO* regulates adipocyte differentiation *in vivo* and that this is the mechanism by which *FTO* affects fat mass. Furthermore, we show that *FTO* acts early in adipogenesis, during mitotic clonal expansion (MCE), to enhance adipocyte number.

## Results

### *FTO* promotes adipogenesis *in vitro*

**Figure 1:**
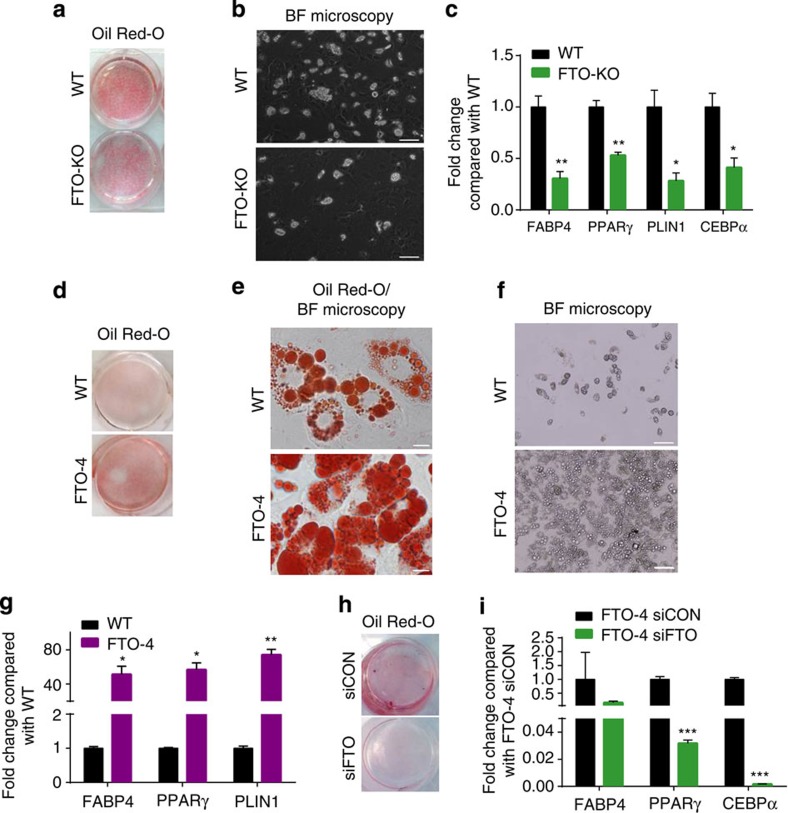
*FTO* overexpression promotes adipogenesis, while *FTO* deletion inhibits adipogenesis *in vitro*. (**a**) Triglyceride uptake (Oil Red-O staining), (**b**) morphology (brightfield microscopy; scale bar, 50 μM) and (**c**) qPCR for mRNA of adipogenic genes in MEFs from WT and *FTO*-KO mice 7 days after adipogenic induction. (**d**) Triglyceride uptake (Oil Red-O staining), (**e**) microscopic images of Oil Red-O stain; scale bar, 20 μM, (**f**) morphology (brightfield microscopy; scale bar, 50 μM, (**g**) qPCR of adipogenic genes 7 days after adipogenic induction in WT and *FTO-4* primary preadipocytes. (**h**) Triglyceride uptake (Oil Red-O staining), (**i**) and qPCR of adipogenic genes, 7 days after onset of adipogenic differentiation in primary preadipocytes from *FTO-4* mice treated with control (black) or *FTO* (green) siRNA. (**a**–**c**) Data represent three biological replicates each with three technical replicates. (**d**–**f**) Data represent three experiments on preadipocytes from one mouse of each genotype. (**g**,**h**) Data represent three experiments on preadipocytes from one mouse. Data presented in graphs represent means±s.e.m.. (**c**,**f**,**h**) Multivariate ANOVA with Bonferroni *post hoc* analysis. *P<0.05; **P<0.01; ***P<0.001.

Mouse embryonic fibroblasts (MEFs) from mice in which *FTO* was either deleted (*FTO-KO*^7^,^8^) or overexpressed (*FTO-4* (ref. 11)) were induced to differentiate into mature adipocytes by treatment with an adipogenic induction cocktail containing dexamethasone, IBMX and insulin. MEFs derived from *FTO-KO* mice exhibited reduced adipogenic capacity, as measured with Oil Red-O staining for triglycerides (Fig. 1a) and light microscopy (Fig. 1b). Quantitative analysis using quantitative PCR (qPCR) showed that this was associated with a reduction in the mRNA levels of *FABP4*, *PPARγ*, *C/EBPα* and *PLIN1* (Fig. 1c), genes that play a critical role in adipogenesis. Protein expression of *FABP4* and *PLIN1* was also lower in *FTO-KO* MEFs than in wild-type (WT) MEFs (Supplementary Fig. 1).

Overexpression of *FTO* produced the opposite result. Following adipogenic induction, primary preadipocytes from the supravascular fraction of gonadal white adipose tissue (gWAT) of *FTO-4* mice exhibited strikingly greater triglyceride accumulation than WT mice (Fig. 1d–f). Expression of the adipogenic genes *FABP4*, *PPARγ* and *PLIN1* was 50-, 55- and 70-fold, respectively, greater in *FTO-4* than in WT preadipocytes (Fig. 1g). Furthermore, protein expression of *FABP4* was higher in *FTO-4* MEFs than in WT MEFs (Supplementary Fig. 2). To confirm that these pro-adipogenic changes were *FTO*-dependent, we knocked down *FTO* by short interfering RNA (siRNA) in primary preadipocytes from gWAT of *FTO-4* mice (Supplementary Fig. 3). This attenuated triglyceride accumulation (Fig. 1h) and significantly reduced adipogenic gene expression (Fig. 1i) when compared with preadipocytes treated with control siRNA.

As it has been proposed that *IRX3* and *RPGRIP1L* are primarily responsible for the enhanced obesity associated with the obesity-related SNPs in *FTO*^12^,^13^, we compared mRNA levels of *FTO*, *IRX3* and *RPGRIP1L* in WT gWAT and MEFs. Levels of *IRX3* and *RPGRIP1L* were considerably lower than that of *FTO* in both gWAT and MEFs of WT mice. Further, *FTO* expression was 13-fold higher than that of *RPGRIP1L* and over 1,000-fold higher than that of *IRX3* in gWAT of WT mice (Supplementary Fig. 4). Importantly, we did not observe any significant differences in *IRX3* or *RPGRIP1L* gene expression in *FTO-4* MEFs (Supplementary Fig. 5a), *FTO-KO* MEFs (Supplementary Fig. 5b) or *FTO-4* gWAT (Supplementary Fig. 5c), when compared with WT MEFs and gWAT of WT mice. These data provide evidence that the effects of *FTO* overexpression and knockdown on adipogenesis are independent of either *IRX3* or *RPGRI1PL*.

### *FTO* regulates MCE in a demethylation-dependent manner

We observed that *FTO* affects adipogenesis by influencing events early in the adipogenic programme. Supplementary Fig. 6 shows that 3 days after adipogenic induction *PPARγ* gene expression was significantly higher in *FTO*-4 MEFs than WT controls, and both *PPARγ* and *FABP4* gene expression were lower in *FTO-KO* MEFs than in WT controls. Expression of *FTO* itself is also regulated during adipogenesis, being stable during the first 48 h after induction and then declining dramatically (Supplementary Fig. 7), as has been shown before^23^,^24^.

**Figure 2:**
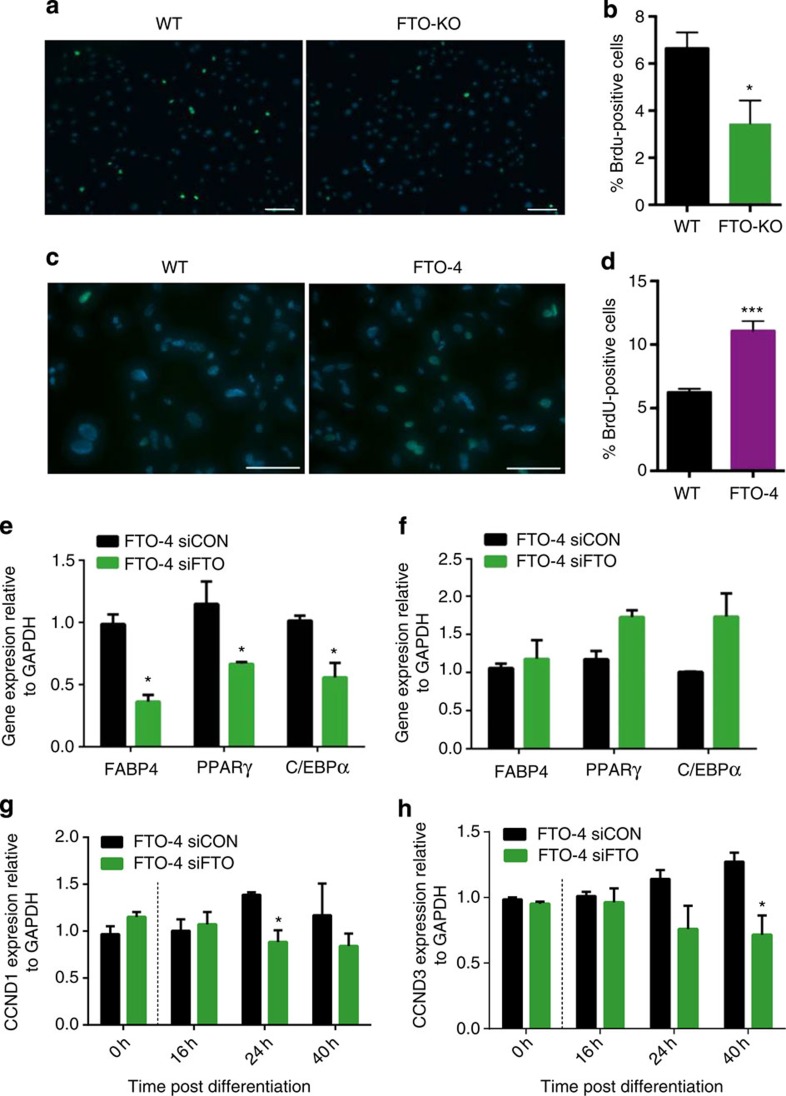
*FTO* promotes adipogenesis via mitotic clonal expansion. (**a**) Representative image showing BrdU incorporation, 24 h after induction of adipogenic differentiation in MEFs from WT and *FTO-KO* mice, BrdU (green), DAPI (blue); scale bar, 100 μM. (**b**) Quantification analysis of BrdU incorporation in WT versus *FTO-KO* MEFs. (**c**) Representative image showing BrdU incorporation, 24 h after induction of adipogenic differentiation in MEFs from WT and *FTO-4* mice, BrdU (green), DAPI (blue); scale bar, 50 μM. (**d**) Quantification analysis of BrdU incorporation in WT versus *FTO-4* MEFs. (**c**,**d**) Data represent six experiments using MEFs derived from one mouse of each genotype. (**e**,**f**) qPCR of mRNA for the indicated genes in *FTO-4* MEFs treated with control (black) or *FTO* (green) siRNA before (**e**) or 48 h after (**f**) adipogenic induction. Data are expressed relative to expression of *GAPDH*. Data represent three experiments each with three technical replicates on MEFs from one mouse. (**g**,**h**) qPCR of mRNA for *CCND1* (**g**) and *CCND3* (H) in *FTO-4* MEFs treated with control (black) or *FTO* (green) siRNA, at 16, 24 and 40 h after induction of adipogenic differentiation. Data represent 1 (0 h) or 4 (16, 24 and 40 h) biological replicates each with three technical replicates. Independent Students’ *t*-test (**c**,**d**), multivariant ANOVA (**e**,**f**), repeated-measures ANOVA (**g**,**h**). *P<0.05; ***P<0.001 against WT (**c**,**d**) or control (**e**–**h**). Data presented in graphs represent means±s.e.m.

We next measured MCE, using 5-bromodeoxyuridine (BrdU) incorporation to assess cell proliferation. Twenty-four hours after induction of adipogenesis, *FTO-KO* MEFs showed less proliferation than WT MEFs (Fig. 2a,b). Conversely, *FTO-4* MEFs displayed greater proliferation than WT MEFs (Fig. 2c,d). These differences do not originate in alterations in cell survival/death, as no differences in cell death were found between WT and *FTO-4* MEFs, or between WT and *FTO-KO* MEFs (Supplementary Fig. 8). Knockdown of *FTO* in *FTO-4* MEFs by siRNA, before stimulation with the adipogenic cocktail, attenuated BrdU incorporation, confirming the central role of *FTO* in adipogenesis (Supplementary Fig. 9).

To determine when *FTO* acts to regulate adipogenesis, we next knocked down *FTO* in *FTO-4* MEFs 48 h after adipogenic induction. While *FTO* knockdown before adipogenic induction reduced the expression of *PPARγ*, *FABP4* and *C/EBPα* (Figs 1c,h and 2e), *FTO* knockdown after MCE had no effect on adipogenic gene expression on day 7 (Fig. 2f). Thus, *FTO* exerts its effects early in adipogenesis, during the MCE phase. To further assess the involvement of *FTO* in MCE, we studied its effects on the expression of *CCND1* and *CCND3*, two cyclin genes that are important for cell cycle progression^25^. Both these genes are upregulated by induction of adipogenesis (Fig. 2g,h). Transfection of *FTO-4* MEFs with *FTO* siRNA led to a significant downregulation of both *CCND1* (Fig. 2g) and *CCND3* (Fig. 2h) at 24 h (*CCDN1*) and 40 h (*CCDN3*) after adipogenic induction when compared with *FTO-4* MEFs transfected with control siRNA. Conversely, overexpression of *FTO* in WT MEFs caused a significant upregulation of *CCND1* expression 24 h following adipogenic induction (Supplementary Fig. 10).

**Figure 3:**
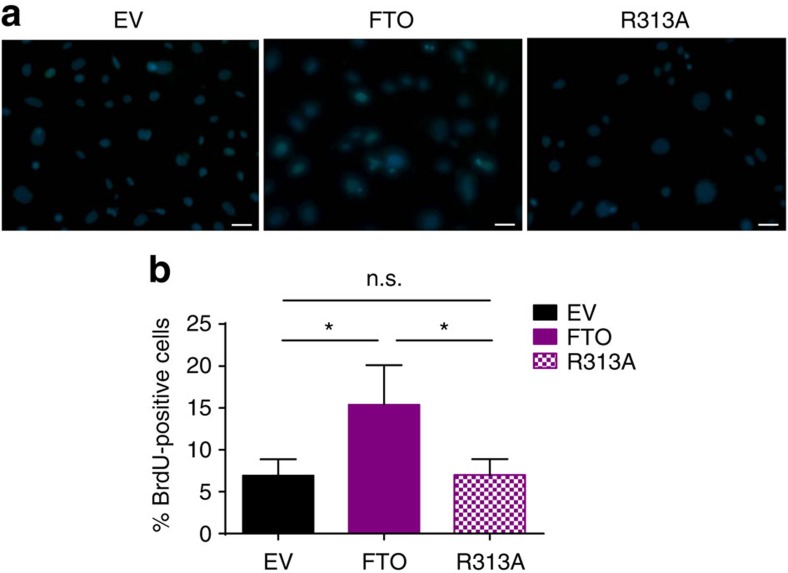
Effect of overexpression of *FTO* and catalytic-null-*FTO* in *FTO-KO* MEFS on BrdU incorporation. (**a**) BrdU incorporation 24 h after adipogenic induction in *FTO-KO* MEFs transfected with empty vector (left), full-length *FTO* (middle) or catalytic-null R313A *FTO* (right); scale bar, 20 μM. (**b**) Quantification of BrdU incorporation in *FTO-KO* MEFs transfected with full-length *FTO* (purple), R313A (catalytic-null) *FTO* (patterned) or empty vector (black) as a control. Results come from four different experiments using MEFs from one mouse. One-way ANOVA, *P<0.05. Data presented in graphs represent means±s.e.m.

We next examined whether the demethylase activity of *FTO* is required for its effects on adipocyte proliferation. We overexpressed WT *FTO* or a catalytically inactive *FTO* (R313A) in *FTO-KO* MEFs and compared BrdU staining 24 h after adipogenic induction. *FTO*-R313A is the mouse equivalent of human R316A, which lacks catalytic activity^17^. Figure 3 shows that MEFS transfected with WT *FTO*, but not with the vector alone, showed enhanced proliferation. In contrast, transfection with *FTO*-R313A did not increase proliferation (Fig. 3). These results demonstrate that intact demethylation activity is necessary for the effect of *FTO* on MCE.

### *FTO* might exert its effects on MCE by acting through RUNX1T1

**Figure 4:**
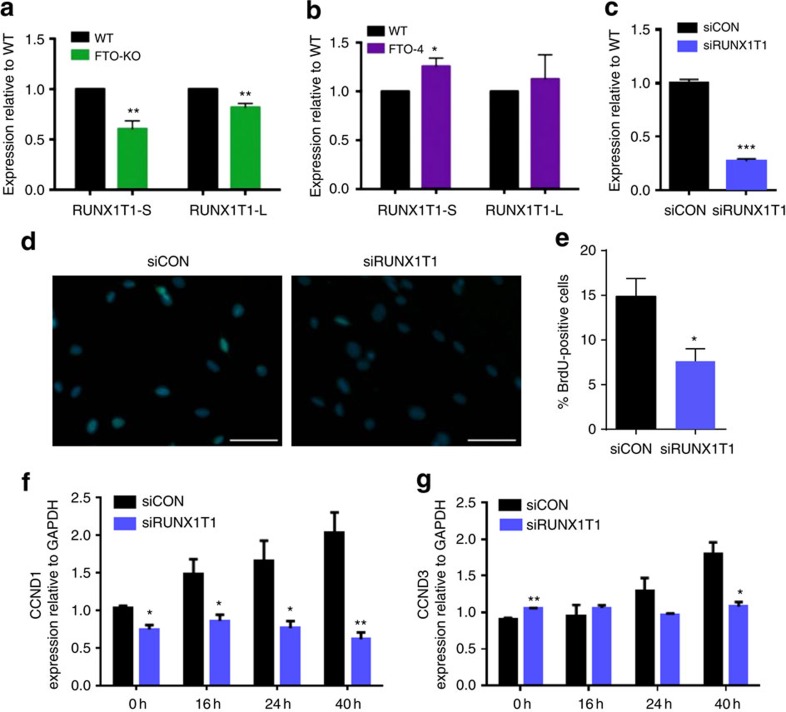
*FTO* regulates RUNX1T1 isoform expression. (**a**,**b**) Expression of the L (496 bp) and S (245 bp) isoforms of RUNX1T1 in WT (black), *FTO-4* (purple) and *FTO-KO* (green) MEFs. Expression was normalized to that of actin, and expressed relative to WT. Data represent three biological replicates each with three technical replicates. (**c**) qPCR of RUNX1T1 mRNA after treatment with control (black) or RUNX1T1 (blue) siRNA. (**d**) BrdU incorporation 24 h after induction of adipogenic differentiation in WT MEFs transfected with control (black) or RUNX1T1 (blue) siRNA; scale bar, 50 μM. (**e**,**f**) qPCR of mRNA for *CCND1* (**e**) and *CCND3* (**f**) in WT MEFs treated with control (black) or RUNX1T1 (blue) siRNA, at 16, 24 and 40 h after induction of adipogenic differentiation. (**c**,**d**) Data represent two biological replicates each with three technical replicates. (**e**,**f**) Data represent three biological replicates each with three technical replicates. Independent Students’ *t*-test (**a**–**d**), repeated-measures ANOVA (**e**,**f**). *P<0.05; **P<0.01; ***P<0.001 against WT (**a**,**b**) or control (**c**–**f**). Data presented in graphs represent means±s.e.m.

It has been suggested that deletion of *FTO* attenuates adipogenesis by inhibition of the pro-adipogenic S isoform of RUNX1T1 (ref. 22). To evaluate this possibility, we first analysed expression of the S and L isoforms of RUNX1T1 in *FTO-4* and *FTO-KO* MEFs. We found that RUNX1T1-L was expressed at much higher levels than RUNX1T1-S in MEFs. Our results revealed a decrease in RUNX1T1-S in *FTO-KO* MEFs (Fig. 4a). However, we observed that *FTO* overexpression was associated with a clear increase in RUNX1T1-S (Fig. 4b). The L isoform of RUNX1T1 was downregulated in *FTO-KO* MEFs, but to a lesser extent than the S isoform, and no difference was observed in RUNX1T1-L when *FTO* was overexpressed. Thus, the S isoform of RUNX1T1 appears to play a greater role in adipogenesis than the L isoform.

We next knocked down RUNX1T1 in WT MEFs using siRNA (Fig. 4c) and compared cell proliferation (BrdU staining) with that found for WT MEFs transfected with control siRNA. Knockdown of RUNX1T1 reduced proliferation, measured 24 h after adipogenic induction (Fig. 4d,e). It also prevented the upregulation of *CCND1* and *CCND3* in response to induction of adipogenesis (Fig. 4f,g). These results provide strong support for a role for RUNX1T1-S in MCE.

Taken together, our results show that *FTO* promotes adipogenesis by activating MCE and suggest that this effect is dependent on its catalytic activity. Furthermore, the data are consistent with the idea that *FTO* mediates its effect by enhancing expression of the S form of RUNX1T1. To examine the physiological relevance of *FTO*-dependent adipogenesis, we next investigated whether this regulation contributes to the increased fat mass observed in mice overexpressing *FTO*.

### Mice overexpressing *FTO* exhibit increased gWAT hyperplasia

Female mice overexpressing *FTO* (*FTO-4*) on a C57BL/6J background gained significantly more weight than WT mice when given a normal chow diet (Supplementary Fig. 11a). These differences became more obvious when mice were fed a high-fat diet (HFD; 45% kcal fat) between 17 and 28 weeks of age (Supplementary Fig. 11a). Differences in body weight between WT and *FTO-4* mice were predominantly due to fat mass (Supplementary Fig. 11b), as lean mass remained similar between groups (Supplementary Fig. 11c). These results confirm previous studies showing that a HFD results in a greater increase in body weight and adiposity in *FTO-4* than in WT mice^11^.

**Figure 5:**
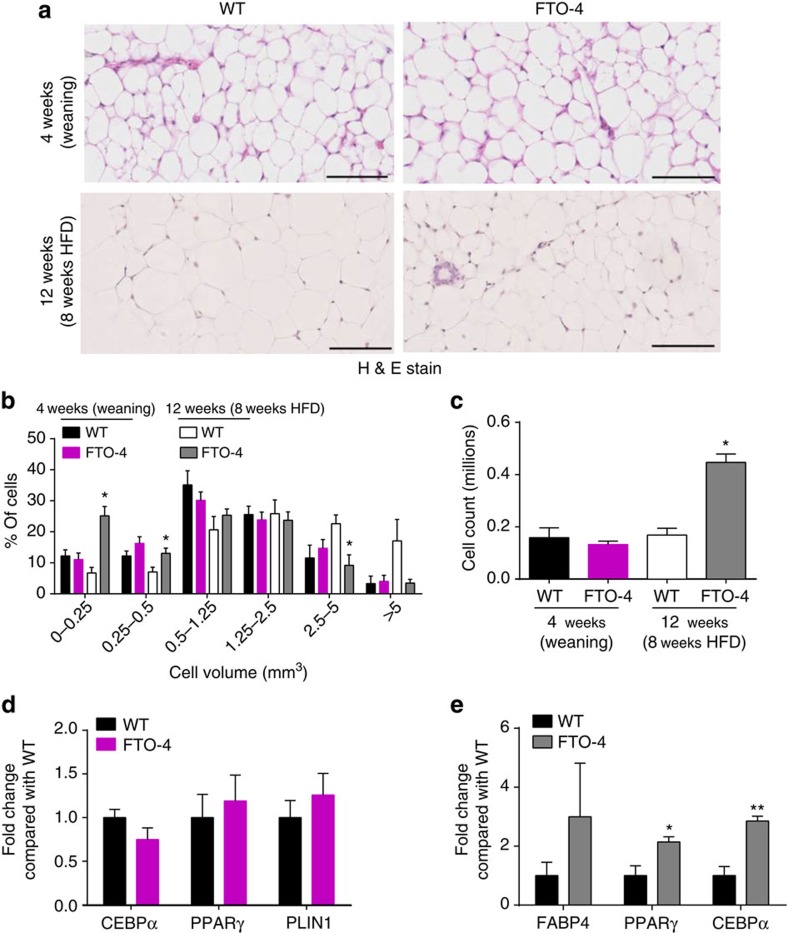
*FTO* overexpression promotes adipogenesis *in vivo*. (**a**) Light microscope images ( × 20 magnification) of haematoxylin/eosin-stained gWAT from WT (left) and *FTO-4* (right) mice after weaning (top, WT, *n*=3, *FTO-4*, *n*=5) and after 8 weeks of HFD following weaning (bottom, WT, *n*=4, *FTO-4*, *n*=3), scale bars, 50 μM. (**b**,**c**) Distribution of adipocyte volume (**b**) and adipocyte number (**c**) in gWAT of WT (black, *n*=4) and *FTO-4* (purple, *n*=3) mice after weaning (filled bars) and after 8 weeks of HFD following weaning (patterned bars). Independent Student’s *t*-test (**c**) or a repeated-measures ANOVA (**b**). *P<0.05; **P<0.01 against WT. (**d**,**e**) qPCR of the indicated genes in gWAT from WT (black) and *FTO-4* (purple) mice after weaning (**d**) or after being fed a HFD for 8 weeks from weaning (**e**). Multivariate ANOVA. *P<0.05; **P<0.01. Data presented in graphs represent means±s.e.m.

To test whether adipose tissue hyperplasia is responsible for the elevated fat mass of *FTO-4* mice, we carried out histological analyses of gWAT of WT and *FTO-4* mice, both at weaning (4 weeks of age) and after 8 weeks of a HFD initiated at weaning (12 weeks of age). At weaning, gWAT from WT and *FTO-4* mice did not differ in morphology when observed with haematoxylin and eosin (H&E) staining (Fig. 5a). After 8 weeks of HFD, however, *FTO-4* mice had smaller but more numerous adipocytes than WT mice (Fig. 5a). Quantitative image analysis of adipocyte volume revealed that as many as 25% of cells in *FTO-4* gWAT had cell volumes below 0.25 mm^3^, compared with 8% of WT gWAT cells after 8 weeks of HFD (Fig. 5b). Analysis of total adipocyte number revealed a significantly greater cell number in *FTO-4* gWAT (0.45 million adipocytes) than WT gWAT (0.17 million adipocytes) after 8 weeks of HFD (Fig. 5c). The greater number of adipocytes in gWAT of *FTO-4* mice given a HFD is consistent with the enhanced proliferation of *FTO-4* MEFs following induction of adipogenesis.

After HFD feeding for 8 weeks, gWAT from *FTO-4* mice also expressed higher levels of *PPARγ* and *C/EBPα* mRNAs than WT mice. No such difference was found immediately after weaning (Fig. 5d,e). There were no significant differences in gWAT depot weights at weaning between WT and *FTO-4* mice, or after 8 weeks of high-fat feeding (Supplementary Fig. 12). The latter may be attributed to the facts that the adipocytes in *FTO-4* mice are not only more numerous but also smaller, and that expansion of adipocyte size is a feature usually observed for HFD feeding of longer than 2 months^26^.

**Figure 6:**
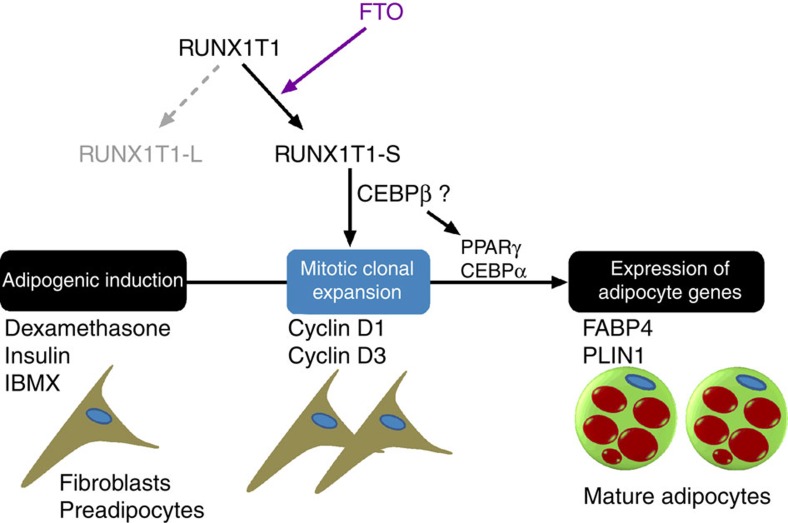
Schematic overview of the proposed role of *FTO* in adipogenesis. *FTO* increases the relative expression of the pro-adipogenic RUNX1T1-S isoform, which stimulates mitotic clonal expansion through stimulating an increase in D-type Cyclin gene expression. This stimulation of mitotic clonal expansion by *FTO* leads to the promotion of adipogenesis, the *de novo* formation of adipocytes from fibroblasts and adipocyte precursors (preadipocytes).

Taken together, our results indicate that *FTO* overexpression is associated with increased adipogenic capacity, through the increased activation of MCE. These effects are potentially mediated through the relative increase in the pro-adipogenic S isoform of RUNX1T1. This is summarized in a schematic diagram (Fig. 6).

## Discussion

Our study provides strong evidence that *FTO* regulates adipogenesis and thereby influences fat mass and body weight. We show that enhanced *FTO* activity increased adipogenesis, whereas decreased *FTO* activity inhibited adipocyte formation, in both MEFs and primary preadipocytes from genetically modified mice. Furthermore, as predicted from these *in vitro* studies, high-fat feeding in mice overexpressing *FTO* induced adipocyte hyperplasia.

Our results demonstrate that *FTO* is a critical regulator of adipogenesis in mice that acts in the early stages of adipogenesis. MCE is a prerequisite for adipocyte differentiation that occurs within 48 h of adipogenic stimulation. During MCE, growth-arrested preadipocytes and MEFs re-enter the cell cycle and undergo two rounds of proliferation. The transcription factor *C/EBPβ* gains the capacity to bind DNA, which leads to the upregulation of *PPARγ* and *CEBPα*, the master regulators of adipogenesis^27^. This is accompanied by initiation of a transcriptional cascade that results in terminal adipogenic differentiation^28^,^29^,^30^ (Fig. 6). We found that *FTO* knockdown inhibits adipogenesis but only before MCE, and that *FTO* overexpression, as expected if it induces MCE, leads to expression of *PPARγ* and *CEBPα*.

Our data support the idea that *FTO* acts by regulating the splicing of the adipogenesis-related transcription factor RUNX1T1 (ref. 22). We show that *FTO* regulates expression of the S isoform of RUNX1T1 in MEFs. In contrast, expression of RUNX1T1-S was reduced in MEFs from *FTO-KO* mice and, importantly, was increased in MEFs from mice overexpressing *FTO*. Furthermore, knockdown of RUNX1T1 in WT MEFs reduced cell proliferation and expression of cell cycle genes during MCE.

Previous studies in 3T3-L1 cells have shown that overexpression of the L isoform of RUNX1T1 impairs adipogenesis^22^,^31^, and, conversely, that overexpression of the S isoform of RUNX1T1 enhances adipogenesis^22^. The effect of RUNX1T1 on adipocyte differentiation and proliferation will therefore be determined by the balance between the L and S isoforms of RUNX1T1. Earlier studies that did not differentiate between the L and S isoforms found that overexpression of RUNX1T1 inhibited adipogenesis by binding of RUNX1T1 to *C/EBPβ* and thereby preventing its DNA-binding activity and the transcriptional cascade that results in adipocyte formation^31^. Given that the L isoform of RUNXT1 is found at much higher levels than the S isoform in 3T3-L1 cells^22^, it seems likely that this effect is due to the predominance of RUNXT1-L. How the S isoform stimulates the adipogenic cascade remains an open question—but an obvious possibility is that it binds to, and activates, *C/EBPβ*. Interestingly, *FTO* has been shown to act as a transcriptional co-activator of *C/EBPβ* (ref. 32).

It is important to recognize that knockdown of RUNX1T1 will result in the loss of both S and L isoforms. Because knockdown leads to reduced cell proliferation, this favours the idea that the effect of the S form is dominant both in MEFs (this paper) and microglia^33^. Our data therefore support the role of the *FTO*/RUNX1T1 interaction in modulating adipogenesis recently reported in ref. 22 and, further, show that this interaction influences the MCE stage of adipogenesis.

While it remains controversial as to whether the obesity-related SNPs in *FTO* act via modulation of *FTO* function or by influencing adjacent genes such as *IRX3* and *RPGRIP1L*, our results provide firm evidence that *FTO* modulates adipogenesis, and thereby fat mass, both *in vitro* and *in vivo*.

Our *in vivo* studies focus on the response to high-fat feeding. Adipose expansion in mice in response to HFD feeding can be produced by both hypertrophy (increased cell size) and hyperplasia (increased cell number), with the relative predominance of each depending on the fat depot^26^. Whereas hypertrophy predominates in the first month of HFD feeding in WT mice, HFD feeding beyond this time results in hyperplasia, which becomes the predominant means by which gWAT expands at 2 months^26^. Although there were no differences in gWAT adipocyte number between WT and *FTO-4* mice at weaning (4 weeks of age), after 8 weeks of a HFD gWAT cell number in *FTO-4* mice was much greater than that of WT mice. Our data further suggest that in *FTO-4* mice, the increase in the size of fat cells may be slightly delayed with respect to WT, as after 2 months of a HFD *FTO-4* adipocytes were smaller, as well as more numerous, than those of WT mice. A previous study has shown greatly increased adipocyte size after 30 weeks of HFD in gWAT from *FTO-4* mice compared with WT mice^11^.

In contrast to a previous study^26^, we did not observe a significant increase in adipocyte number in response to 2 months of high-fat feeding in WT mice. This difference may be attributable to differences in the fat content of the different diets: 45% kcal as fat in our study compared with 60% kcal as fat in the previous study^26^. Furthermore, in our study, mice were started on a HFD at 4 weeks of age compared with 10 weeks of age in the previous study. We cannot rule out the possibility that gWAT hyperplasia might also occur on a chow diet, particularly in light of previous studies showing increased adiposity in *FTO-4* versus WT mice maintained on a chow diet^11^.

Although it is still unclear whether any of the known intronic SNPs in *FTO* can modulate *FTO* expression, our data have implications for humans carrying gain-of-function variants in *FTO*. They suggest that such individuals might show enhanced adipogenesis, especially on a diet high in fat. Furthermore, fat cells induced by a HFD can be expected to remain even after reverting to a less calorific diet. This would lead to an enhanced storage capacity and a greater propensity for increased fat mass and body weight when energy consumption exceeds energy expenditure.

## Methods

### Ethical statement

Animal studies were conducted in accordance with the UK Animals (Scientific Procedures) Act (1986). All mice were maintained in accordance with the UK Home Office Welfare guidelines and project licence restrictions. All studies were approved by the local Animal Welfare and Ethical Review Body at MRC Harwell, under the ethical guidelines issued by the Medical Research Council (Responsibility in the Use of Animals for Medical Research, July 1993).

### Mouse studies

Mice constitutively expressing two additional copies of *FTO* in all tissues on a C57BL/6J background (*FTO-4*) were generated by insertion of the *FTO* cDNA into a pCAGGs-STOP-EGFP-ROSA-TV plasmid (downstream of the STOP cassette). Targeted stem cell injections were given to C57BL/6J blastocysts to generate chimeras that transmitted the targeted allele when crossed to C57BL/6J mice. F1 mice were crossed to a line carrying the β-actin-Cre recombinase (Jackson Laboratory, stock name Tg(ACTA1-cre)79Jme/J) on a C57BL/6J background, and the offsprings were backcrossed again to C57BL/6J to remove Cre recombinase. These mice were then intercrossed in multiple different matings to generate the test populations. WT C57BL/6J littermates were used as controls. All experiments were carried out on female mice maintained in a temperature- (21±2 °C) and humidity (55±10%)-controlled room on a 12:12 light dark cycle (light 0700–1900, h). Mice had *ad libitum* access to water (9–13 p.p.m. chlorine) and food (SDS Rat and Mouse No. 3 Breeding diet (RM3) containing 11.5 kcal% fat, 23.9 kcal% protein and 61.6 kcal% carbohydrate). When indicated, mice were maintained on a HFD (D12451, Research Diets, New Brunswick, NJ) containing 45 kcal% fat, 20 kcal% protein and 35 kcal% carbohydrate. Body composition was measured with an Echo MRI whole-body composition analyzer (Echo Medical System, Houston, TX).

### Histology

Serial sections of fixed gWAT of WT and *FTO-4* mice were stained with H&E. ImageJ was used to determine the mean cell volume per section. Total adipocyte number was determined by using the equation: *n*=*m* (depot mass g)/*P* (density of adipose 0.915 g cm^−3^) × volume (cm^3^) as previously described^34^.

### MEFs

To obtain MEFs, timed matings of FTO-3 mice (to obtain *FTO-4* embryos) or heterozygous *FTO-KO* mice (to obtain *FTO-KO* embryos) were set up. WT littermate embryos were used as controls. Mothers were killed at E12.5−E14.5 and embryos collected. Following removal of the head, liver and blood clots, individual embryos were dissociated in 0.25% Trypsin (Gibco, Paisley, Scotland) and subsequently triturated. MEFs were cultured at 37 °C and 5% CO_2_ in DMEM (Gibco) supplemented with 10% fetal bovine serum and 1 × penicillin/streptomycin (Gibco). The heads of the embryos were used for DNA extraction and genotyping.

### Primary adipocyte culture

Primary adipocytes were isolated from dissected gWAT of 12-week-old mice and digested with collagenase (Sigma) in Krebs-Ringer HEPES buffer at 37 °C for 1 h. The digested tissue was filtered, and was then centrifugated to obtain the supravascular fraction, which was resuspended and cultured in DMEM (Gibco) supplemented with 10% fetal bovine serum and 1 × penicillin/streptomycin (Gibco). Adipogenic differentiation was induced by supplementing the tissue culture medium with 250 μM IBMX, 0.1 μM dexamethasone and 0.5 μg ml^−1^ insulin for 4 days. After this period, the culture medium was supplemented with insulin only.

Knockdown of *FTO* or RUNX1T1 in cell lines was achieved using Lipofectamine RNAiMAX (Invitrogen, CA, USA) using siRNA directed against mouse *FTO* (5′-CCUGCGAUGAUGAAGUGGACCUUAA-3′ (Invitrogen)), mouse RUNX1T1 (no. 161027, Invitrogen) and stealth RNA interference siRNA Negative Control Medium GC (Invitrogen) as a control. To overexpress *FTO*, cells were transfected using Lipofectamine2000 (Invitrogen) with either a pcDNA3.1 vector containing full-length mouse *FTO* cDNA, R313A full-length mouse *FTO* cDNA or an empty pcDNA3 vector as a control. Cell viability after transfection was assessed using the LIVE/DEAD viability/cytotoxicity kit (Life Technologies).

### Oil Red-O staining

Cells were washed with PBS and then fixed in 10% buffered formalin for 1 h at room temperature. After washes in PBS, cells were stained for 30 min at room temperature with a filtered Oil Red-O (Sigma) solution (0.5% Oil Red-O in isopropyl alcohol), washed again in PBS and visualized under an inverted microscope (Olympus).

### BrdU analysis

To examine proliferation, a BrdU incorporation assay was performed using a FLUOS *In situ* detection kit (Roche) on at least six technical replicates. BrdU was added 24 h after the onset of adipogenic differentiation for 30–45 min. Immunodetection of BrdU was carried out following the manufacturer’s instructions. Cells were also stained with 4,6-diamidino-2-phenylindole (DAPI) to enable nuclear detection. A blinded analysis of percentage of BrdU-positive cells (of total number of cells assessed with DAPI) was performed with ImageJ.

RNA extraction, RT–PCR experiments, protein extraction and western blot analysis were carried out as described before^11^. Expression of the two different isoforms of RUNX1T1 was measured as described in ref. 22.

### RNA extraction and cDNA synthesis

Total RNA from MEFs, primary adipocytes and dissected fat pads was extracted using an RNeasy Mini Plus Kit (Qiagen, USA) according to the manufacturer’s protocol. The RNA concentration was measured with a NanoDrop spectrophotometer (Thermo Scientific), and 1 μg of RNA was reverse-transcribed using the High Capacity cDNA Reverse Transcription kit (Applied Biosystems).

### RT–PCR analysis

Gene expression was analysed using the TaqMan system (all probes and reagents from Life Technologies) on a ABI Prism 7700 Sequence Detection System (Perkin Elmer). Samples were measured in duplicate and gene expression was normalized to the expression of glyceraldehyde-3-phosphate dehydrogenase (*Gapdh*).

### Western blot analysis

MEFs were harvested using the Qiagen kit with protease inhibitors. Samples were kept on ice for at least 20 min, after which nonsoluble material was pelleted at 20,000*g* for 20 min at 4 °C. The supernatant was removed and protein concentrations were estimated using the Bio-Rad Dc Protein assay.

For western blot analysis, 20 μg of the protein lysates were run on 4–12% Bis-Tris SDS–PAGE gels (NuPAGE Novex, Invitrogen) and transferred to nitrocellulose membranes. Membranes were blocked for 1 h at room temperature in 5% milk in TBS-T, and incubated overnight at 4 °C with primary antibodies to *FABP4* (Cell Signaling), *PLIN1* (Cell Signaling) and HSC70 (Abcam, ab19136) as an internal control. Blots were washed in TBS-T and incubated with secondary antibody (horseradish peroxidase (HRP)-anti-rabbit IgG for FABP4 and PLIN1, HRP-anti-rat IgG for HSC70, GE Healthcare) for 1 h at room temperature. After washing, bands were visualized with enhanced chemiluminescence reagent (SuperSignal West Pico, Pierce) and X-Ray film. Protein density was analysed using the ImageJ software (National Institutes of Health, USA). All primary antibodies used were at 1:1000, and all secondary antibodies were at 1:2500 dilutions.

### Data analysis

All experiments were conducted on at least three biological replicates, each of which had at least three technical replicates, unless otherwise stated. All values are expressed as mean±s.e.m. Unless otherwise stated, qPCR data are normalized to GAPDH and expressed relative to WT (or control). Statistical analysis was performed using the IBM SPSS software (version 20) using either an independent Student’s *t*-test or a multivariate analysis of variance (ANOVA) with Bonferroni *post hoc* analysis. Parameters measured over multiple time points were analysed with a repeated-measures ANOVA with time as a within-subject factor. In case of a significant group or time × group interaction, a multivariate ANOVA was run per time point to identify statistically significant differences. Statistical significance was taken as *P*<0.05.

## Additional information

**How to cite this article:** Merkestein, M. *et al.* *FTO* influences adipogenesis by regulating mitotic clonal expansion. *Nat. Commun.* 6:6792 doi: 10.1038/ncomms7792 (2015).

## Supplementary Material

Supplementary Information Supplementary Figures 1-12
