# Supplementary material for: FTO influences adipogenesis by regulating mitotic clonal expansion
Source: Nat Commun. 2015 Apr 17;6:6792. doi: 10.1038/ncomms7792 (PMC4410642; doi:10.1038/ncomms7792)

A

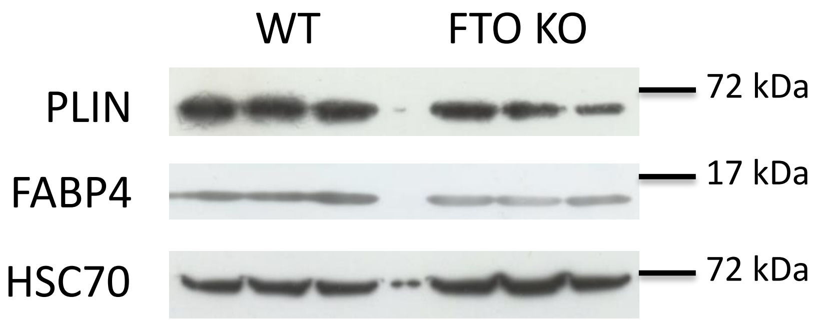

B

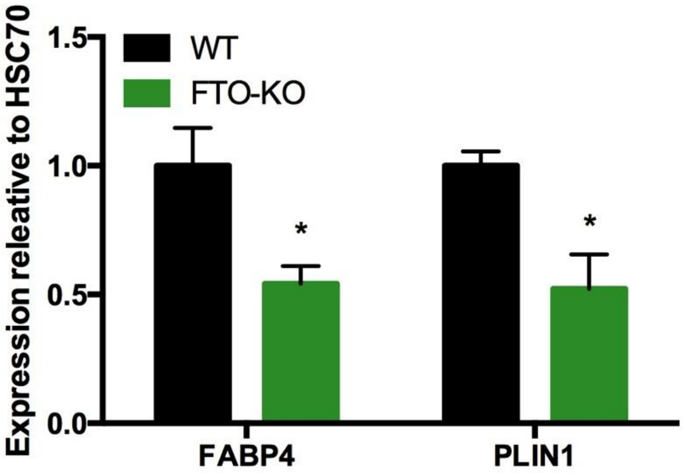

Supplementary Figure 1. Adipogenic protein expression in WT and KO MEFs after 7 days of adipogenic differentiation.

Western blots of PLIN1 and FABP4 from WT and FTO-KO MEFs 7 days after adipogenic induction. The housekeeping protein HSC70 (heatshock protein 70) is included as a loading control. Replicates are different experiments from the same batch of MEFs.B. Quantitative expression (by densitometry) of PLIN1 and FABP4 protein measured in WT (black) and FTO-KO (green) MEFs 7 days after adipogenic induction. Protein expression was expressed relative to HSC70 and then normalised to WT. Replicates are different experiments from the same batch of MEFs (n=3). Differences were analysed using a multivariate ANOVA with Bonferroni post-hoc testing, * $p<0.05$. Graph represents mean±SEM

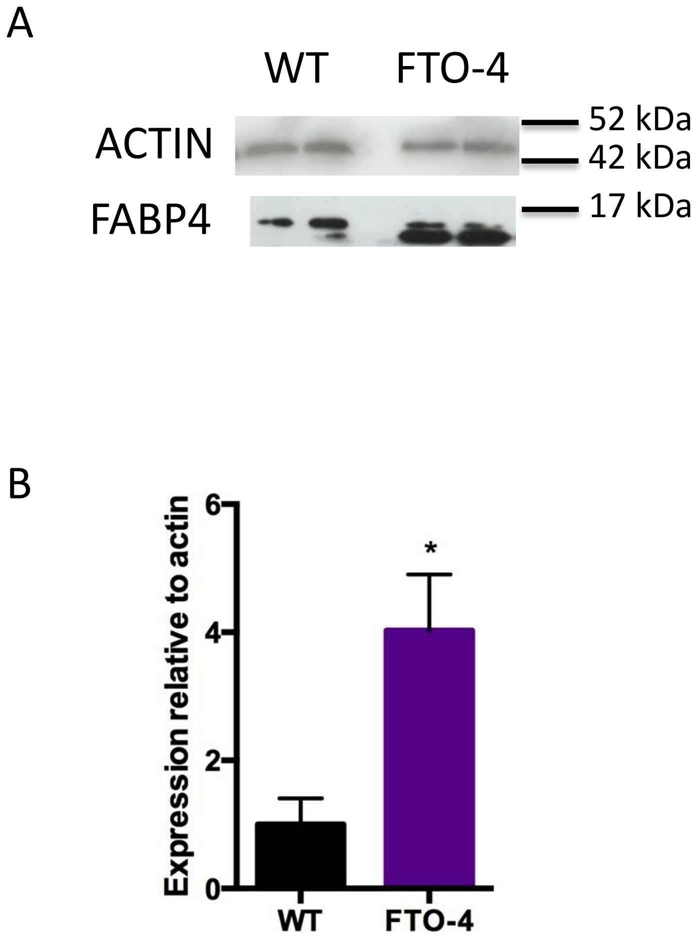

Supplementary Figure 2. Adipogenic protein expression in WT and FTO-4 MEFs after 7 days of adipogenic differentiation.

A. Western blots of FABP4 and actin from WT and FTO-4 MEFs, 7 days after adipogenic induction. Actin was included as a loading control. Replicates are different experiments from the same batch of MEFs.B. Protein expression of FABP4 measured in WT (black) and FTO-4 (purple) MEFs 7 days after adipogenic induction. Protein expression was expressed relative to actin and then normalised to WT. Replicates are different experiments from the same batch of MEFs (n=2). Differences were analysed using an independent Student's t-test, * $p<0.05$. Graph represents mean±SEM

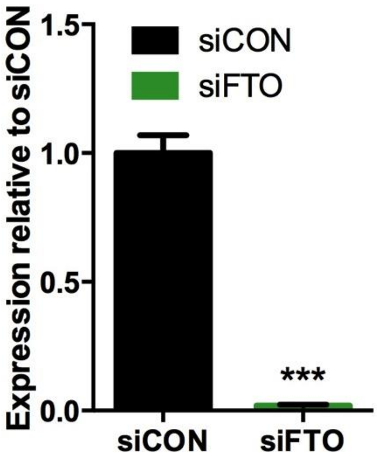

Supplementary Figure 3. Confirmation of FTO knockdown in primary adipocytes derived from FTO-4 mice.

Quantitative PCR of FTO mRNA in FTO-4 MEFs following transfection with control (black) or FTO siRNA (green). Replicates (n=3) are different experiments from the same batch of primary adipocytes (derived from one mouse). Statistical significance was analysed with Student's t-test, *** $p<0.001$. Graph represents mean±SEM.

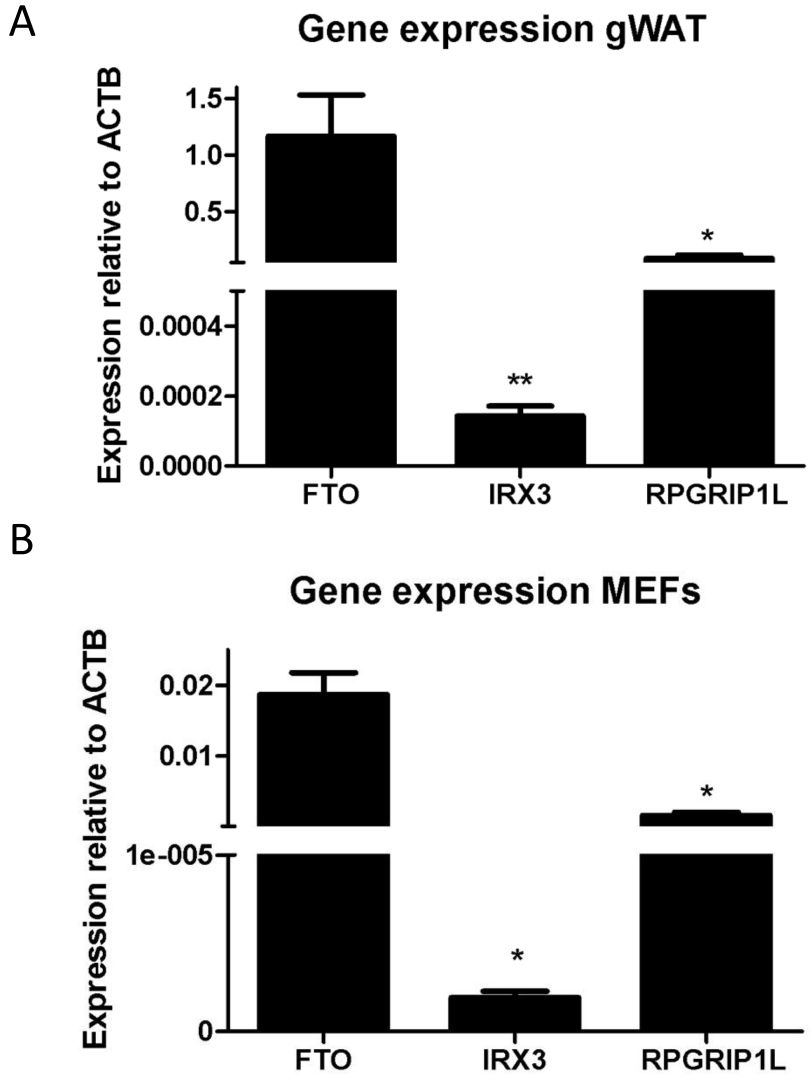

Supplementary Figure 4. Expression levels of *Fto, RPGRIP1L* and *IRX3* mRNAs in gWAT and MEFs from WT mice.

Quantitative PCR of *Fto*, *RPGRIP1L* and *IRX3* mRNAs measured in gWAT (A) and MEFs (B) from WT mice. Data are expressed relative to expression of the housekeeping gene β-actin (ACTB). A, n=4 mice. B, n=3 different experiments using MEFs derived from 3 different mice. Statistical significance was analysed using a oneway ANOVA against *RPGRIP1L* and *IRX3*, * $p<0.05$; ** $p<0.01$. Graph represents mean±SEM.

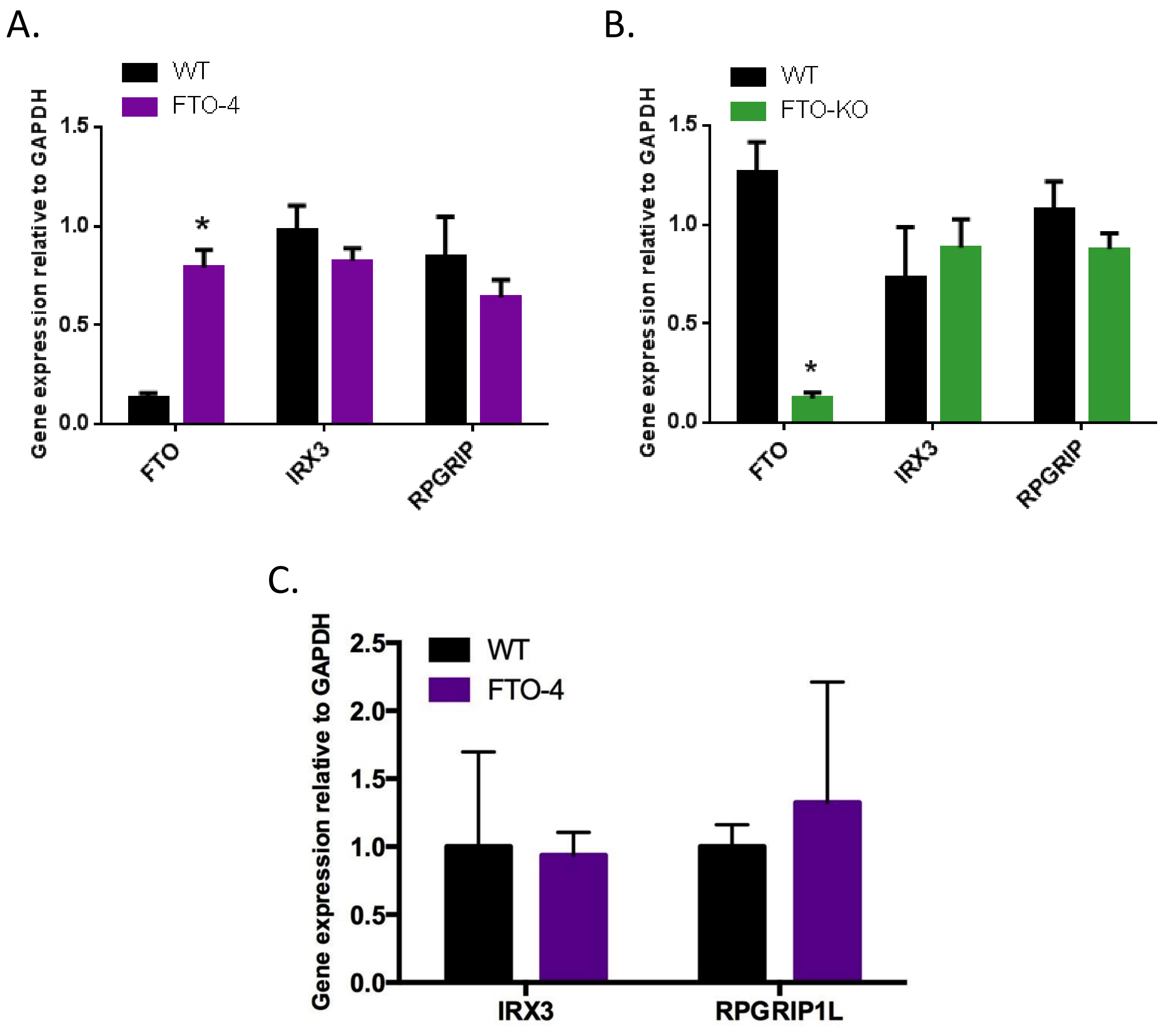

Supplementary Figure 5. Expression levels of *Fto, RPGRIP1L* and *IRX3* mRNAs in gWAT of WT and FTO-4 mice and WT, FTO-KO and FTO-4 MEFs.

Quantitative PCR of *Fto, RPGRIP1L* and *IRX3* mRNAs measured (A) in WT (black, n=2) and FTO-4 (purple, n=5) MEFs; (B) WT (black, n=3) and FTO-KO (green, n=3) MEFs; and (C) gWAT from WT (black, n=3) and FTO-4 (purple, n=3) mice. N numbers represent biological replicates (MEFs derived from different embryos). Statistical significance was analysed by multivariate ANOVA comparing expression of *Fto, RPGRIP1L* and *IRX3* in WT versus FTO-4 or FTO-KO, * $p<0.05$. Graphs represent mean±SEM.

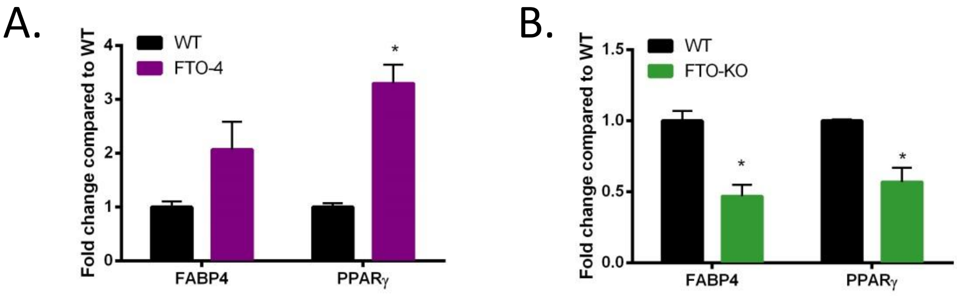

Supplementary Figure 6. Adipogenic gene expression in FTO-KO and FTO-4, and their respective WT MEFs, 3 days after induction of adipogenesis.

qPCR of FABP4 and PPARγ mRNAs from WT (black), FTO-4 (purple) and FTO-KO (green) MEFs 3 days after induction of adipogenic differentiation. n=3 different experiments using MEFs derived from one mouse. Multivariate ANOVA with Bonferroni correction, * $p<0.05$ against WT. Graphs represent mean±SEM.

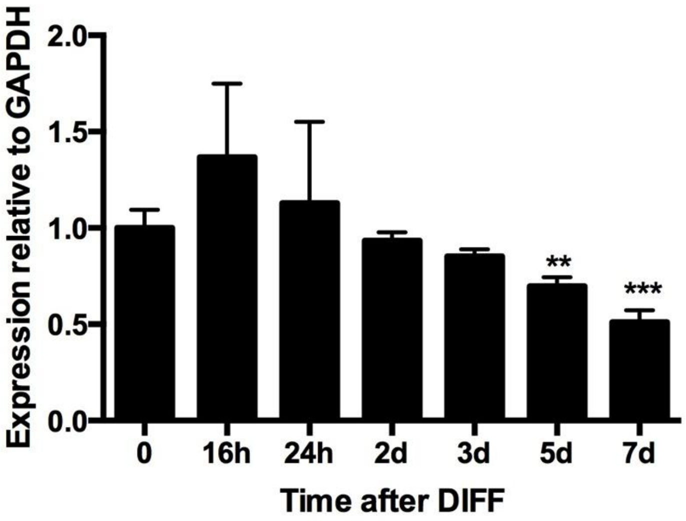

Supplementary Figure 7. *Fto* expression during differentiation in MEFs.

Fto mRNA expression levels measured by qPCR in WT MEFs before (Day 0) and during adipogenic differentiation (16 and 24 hours, 2,3, 5 and 7 days). Data from the 0, 16h and 24h time points consist of data from 2 different batches of MEFs each with 3 technical replicates, the other time points indicate 3 different experiments using MEFs derived from one mouse. Repeated measures ANOVA followed by comparison of all time points to Day 0 with a Dunnett's post-hoc test, and stars indicate significantly different from time point 0, **$p<0.01$; ***$p<0.001$. Graph represents mean±SEM.

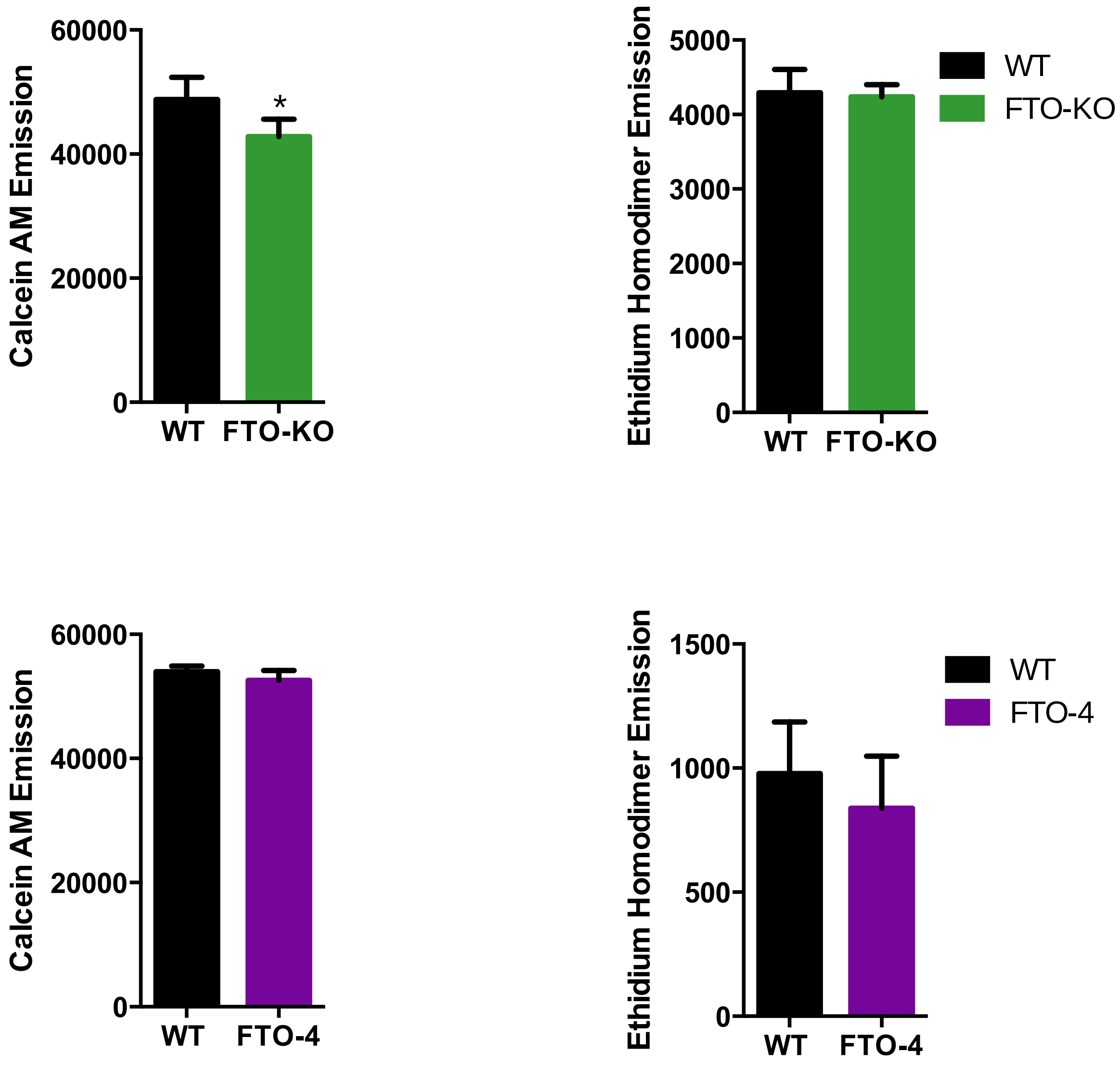

Supplementary Figure 8. Cell viability assay in WT, FTO-KO and FTO-4 MEFs 24 hours after adipogenic induction.

Number of live (left, Calcein EM emission) and dead (right, ethidium homodimer emission) cells 24 hours after adipogenic induction in WT (black), FTO-4 (purple) and FTO-KO (green) MEFs. Data are a minimum of 2 biological replicates, with each 5 technical replicates. Student’s t-test, * $p<0.05$ against WT. Graphs represent mean±SEM.

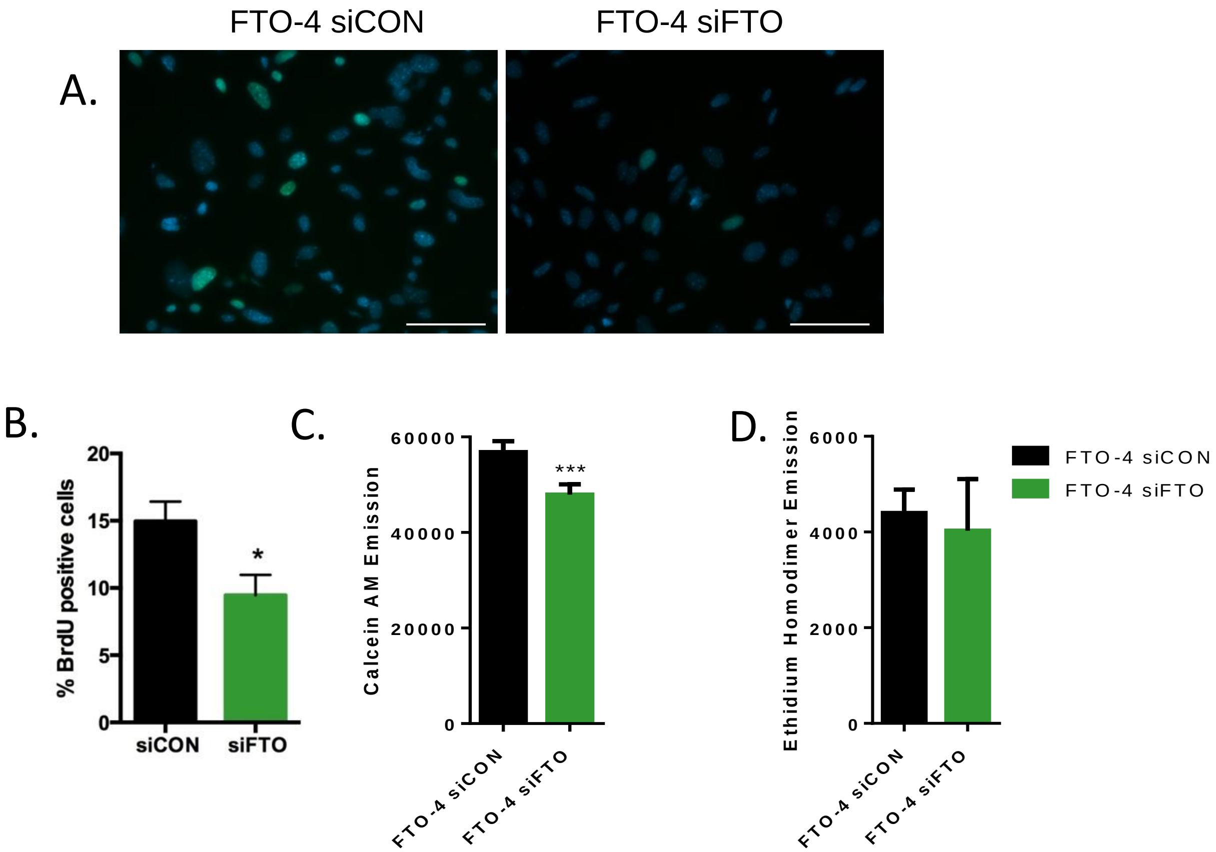

Supplementary Figure 9. BrdU incorporation and cell viability assay 24 hours after adipogenic induction in FTO-4 MEFs treated with FTO siRNA.

A. BrdU incorporation 24 hours after induction of adipogenic differentiation in MEFs from FTO-4 mice treated with control siRNA (left) or FTO siRNA (right). Cells were stained for BrdU (green) and nuclei visualised with DAPI (blue), scale bar, 50μM. B. Quantification of BrdU incorporation in FTO-4 MEFs treated with FTO siRNA (green) or control siRNA (black). Data represent 6 experiments carried out in MEFs derived from one mouse. C, D. Number of live (C, Calcein EM emission) and dead (D, ethidium homodimer emission) cells 24 hours after adipogenic induction in FTO-4 MEFs treated with control siRNA (black) and FTO siRNA (green). Student's t-test, ***p<0.001, * p<0.05 against FTO-4 siCON. Graphs represent mean±SEM.

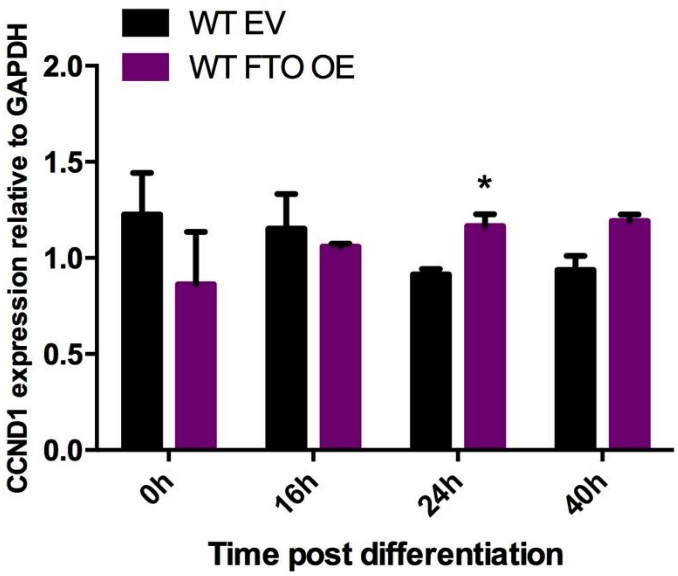

Supplementary Figure 10. CCND1 expression after induction of adipogenic differentiation in WT MEFs that overexpress Fto.

Quantitative PCR of CCND1 mRNA 16, 24 and 40 hours after induction of adipogenic differentiation in WT MEFs transfected with a vector expressing full-length FTO (FTO OE, purple) or empty vector (EV, black) as a control. Data represent 3 different experiments using MEFs derived from one mouse. Multivariate ANOVA comparing empty vector to FTO overexpression at each time point, * $p<0.05$. Graph represents mean±SEM.

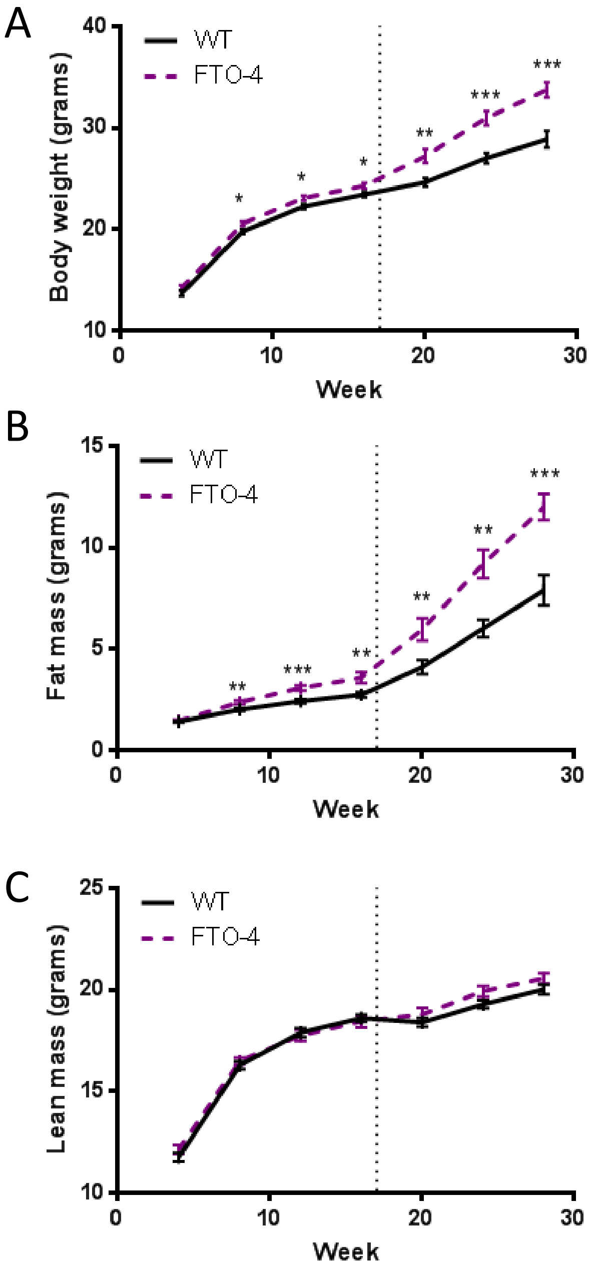

Supplementary Figure 11. FTO overexpression leads to increased body weight and adiposity.

Body weight (A), fat mass (B), and lean mass (C) in FTO-4 mice (n=22, purple) and wildtype (n=20, black) female C57BL/6J mice during chow and high-fat feeding. Animals were maintained on chow from weaning until 17 weeks, at which time they were given a high-fat diet (as indicated by the dotted vertical line) until completion of the study at 28 weeks. Note that the y-axis starts at 10 grams for Supplementary Figures S12A and S12C. Repeated-measures ANOVA, *p<0.05; **p<0.01; ***p<0.001 against WT.Graphs represent means±SEM.

	4 wks old (weaning)	12 wks old (8 wks HFD)
WT	0.078±0.007 mg	0.335±0.05 mg
FTO-4	0.075±0.002 mg	0.413±0.07 mg

Supplementary Figure 12. Weights of gWAT depots of WT and FTO-4 mice after weaning or following an 8-week HFD.

Weights of gWAT depots of WT and FTO-4 mice at 4 weeks of age after weaning (WT, n=3; FTO-4, n=5) or at 12 weeks old following an 8-week HFD initiated after weaning (WT, n=4; FTO-4, n=3). Student’s t-test. Data represent means±SEM.

Original western blot images

Supplementary Figure 1a

PLIN1

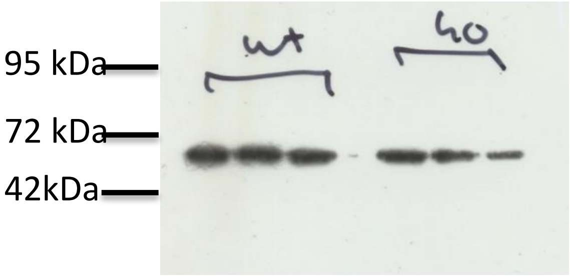

FABP4

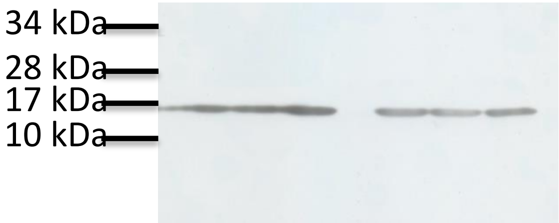

HSC70

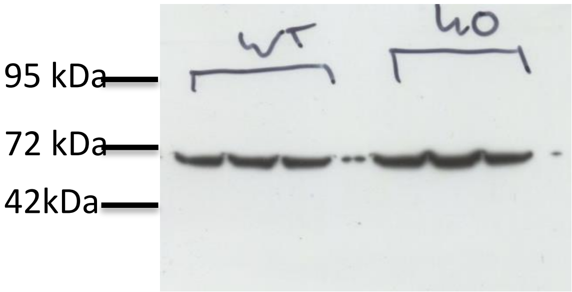

Supplementary Figure 2a

ACTIN

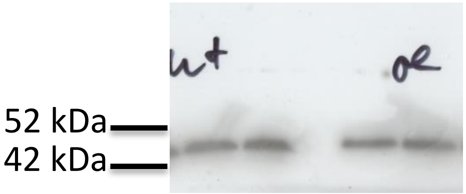

FABP4

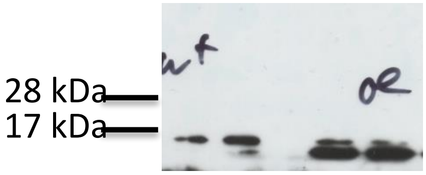

Supplement: Supplementary Information — Supplementary Figures 1-12 [file ncomms7792-s1.pdf]
